# Smoke-Free Laws and Hazardous Drinking: A Cross-Sectional Study among U.S. Adults

**DOI:** 10.3390/ijerph14040412

**Published:** 2017-04-13

**Authors:** Nan Jiang, Mariaelena Gonzalez, Pamela M. Ling, Kelly C. Young-Wolff, Stanton A. Glantz

**Affiliations:** 1 Department of Population Health, School of Medicine, New York University, New York, NY 10016, USA; 2 Center for Tobacco Control Research and Education, University of California, San Francisco, CA 94143, USA; pling@medicine.ucsf.edu (P.M.L.); Stanton.Glantz@ucsf.edu (S.A.G.); 3 School of Social Sciences, Humanities & Arts, University of California, Merced, CA 95343, USA; mgonzalez82@ucmerced.edu; 4 Division of Research, Kaiser Permanente Northern California, Oakland, CA 94612, USA; kelly.c.young-wolff@kp.org

**Keywords:** smoking, alcohol, hazardous drinking, smoke-free law

## Abstract

Tobacco and alcohol use are strongly associated. This cross-sectional study examined the relationship of smoke-free law coverage and smoke-free bar law coverage with hazardous drinking behaviors among a representative sample of U.S. adult drinkers (*n* = 17,057). We merged 2009 National Health Interview Survey data, American Nonsmokers’ Rights Foundation U.S. Tobacco Control Laws Database, and Census Population Estimates. Hazardous drinking outcomes included heavy drinking (>14 drinks/week for men; >7 drinks/week for women) and binge drinking (≥5 drinks on one or more days during past year). Chi-square tests compared hazardous drinking by sociodemographic factors. Multivariable logistic regression models were used to examine if smoke-free law and bar law coverages were associated with hazardous drinking, controlling for sociodemographics and smoking status. Subset analyses were conducted among drinkers who also smoked (*n* = 4074) to assess the association between law coverages and hazardous drinking. Among all drinkers, smoke-free law coverage was not associated with heavy drinking (adjusted odds ratio (AOR) = 1.22, 95% confidence interval (CI) = 0.99–1.50) or binge drinking (AOR = 1.09, 95% CI = 0.93–1.26). Smoke-free bar law coverage was also found to be unrelated to hazardous drinking. Similar results were found among those drinkers who smoked. Findings suggest that smoke-free laws and bar laws are not associated with elevated risk for alcohol-related health issues.

## 1. Introduction

The paired use of tobacco and alcohol increases the risk for a variety of diseases, including cancers of mouth, throat, and upper aerodigestive tract [1,2,3]. However, the co-occurrence of smoking and drinking is common [4,5,6]. Smokers are more likely to drink alcohol than non-smokers [7,8,9,10], and more alcohol drinkers smoke cigarettes than non-drinkers [8,11,12]. Smokers are more likely to meet the DSM-IV criteria (Diagnostic and Statistical Manual of Mental Disorders, Fourth Edition) for alcohol use disorders (AUD) [13], and people with AUD have a higher rate of nicotine dependence than the general population [14].

Smoke-free laws are effective in protecting non-smokers from secondhand smoke [15,16,17], reducing smoking rates and cigarette consumption [18,19,20,21,22,23], and increasing smoking cessation [22,23,24,25]. Stronger smoke-free law coverage is associated with lower odds of current (past 30-day) smoking [26,27]. Recognizing the importance of smoke-free legislation, one of the *Healthy People 2020* objectives is to enact 100% smoke-free laws in public places and worksites [28]. Given the strong relationship between smoking and drinking, smoke-free laws may not only reduce cigarette smoking but also change alcohol use patterns.

However, very few studies have examined the effect of smoke-free laws on alcohol consumption or AUD, and the findings of existing research are inconsistent. Two longitudinal studies found that smoke-free bar laws did not change the frequency and/or amount of alcohol use overall, but reduced drinking quantity among moderate drinkers and heavy smokers [29,30]. Another longitudinal study concluded that state-level smoke-free bar and restaurant laws were associated with increased AUD remission (defined as meeting the AUD criteria at the baseline but not in the follow-up), especially among males, and less AUD onset (defined as meeting the AUD criteria in the follow-up but not at the baseline) among females [31]. However, these longitudinal studies assessed the smoke-free bar and/or restaurant laws only and did not examine the effect of smoke-free laws that cover other important venues (e.g., private and public worksites). Two repeated cross-sectional studies have examined the relationship of comprehensive smoke-free laws and alcohol use. One study found that stronger statewide smoke-free law coverage (in private workplaces, restaurants, and bars) was associated with significant reduction in state per capita alcohol consumption [32]. The other study concluded that stronger state-level smoke-free law coverage (in private and government workplaces, restaurants, bars, health-care facilities, grocery stores, malls and hotels) was associated with reduced alcohol use in females only [33], but it did not account for local-level laws which might be stronger than the state laws.

Given the limited evidence regarding the relationship of smoke-free laws with alcohol use, we conducted this study to examine the association between smoke-free law coverage (which considers the smoke-free law coverage in public workplaces, private workplaces, restaurants and bars, as well as the proportion of county population covered by smoke-free laws targeting each of the four venues [34,35]) and hazardous drinking behaviors among U.S. adult drinkers and among drinkers who also reported current smoking. We also examined the association between smoke-free bar law coverage and hazardous drinking behaviors.

## 2. Materials and Methods

### 2.1. Data Source

We used county Federal Information Processing Standards (FIPS) codes to merge three databases: 2009 National Health Interview Survey (NHIS) restricted data, 2009 American Nonsmokers’ Rights Foundation (ANRF) U.S. Tobacco Control Laws Database, and 2009 Census Population Estimates [34]. An FIPS county code consists of two digits of state code and three digits of county code within the state. The code provides a unique identification of counties and county equivalents within the U.S. The NHIS is a cross-sectional household interview survey among a representative sample of the noninstitutionalized civilian U.S. population. A total of 27,731 respondents aged ≥18 years completed the adult core questionnaires with a response rate of 65.4% [36]. Our study was limited to adult drinkers (*n* = 17,057). Institutional review board approval is not required for this study, because it is a secondary analysis of de-identified NHIS data.

### 2.2. Dependent Variables

Alcohol consumption was measured by three questions: “In any one year, have you had at least 12 drinks of any type of alcoholic beverage?” “In your entire life, have you had at least 12 drinks of any type of alcoholic beverage?” “In the past year, how many days per week, per month or per year did you drink any type of alcoholic beverage?” Consistent with the Centers for Disease Control and Prevention’s (CDC) definition [36,37], respondents who reported 12 or more drinks in their lifetime and at least one drink in the past year were defined as drinkers.

Hazardous drinking, which included heaving drinking and binge drinking, was assessed among drinkers. Following National Institute on Alcohol Abuse and Alcoholism (NIAAA) guidelines [38], respondents who consumed more than gender-specific weekly limits for alcoholic drinks (>14 drinks/week for men; >7 drinks/week for women) were defined as heavy drinkers. Binge drinking was measured by asking, “In the past year, on how many days did you have five or more drinks of any alcoholic beverage?” Respondents who reported consuming five or more drinks on at least one day during the past year were defined as binge drinkers. Though NIAAA defines binge drinking as consuming five or more drinks for men and four or more drinks for women in about 2 h [39], many studies [40,41,42,43,44] and national health surveillance surveys (e.g., Youth Risk Behavior Surveillance Survey [45] and National Survey on Drug Use and Health [46]) used a universal cutoff point of five drinks for both men and women and phrased the question as “in an occasion” or “in a row” while measuring binge drinking behavior.

### 2.3. Independent Variables

As described in detail elsewhere [34,35], linking ANRF Tobacco Control Database and Census Population Estimates database yielded smoke-free law and bar law coverage scores for each county. We used the FIPS county code to merge smoke-free law and bar law coverage scores with the NHIS data. The smoke-free law coverage score is a continuous variable which takes into account two factors: (1) the venues (i.e., public and private workplaces, restaurants and bars) covered by 100% smoke-free laws in each county, and (2) the proportion of county population covered by 100% smoke-free laws targeting each of the four venues. The smoke-free law coverage score ranges from 0 to 1, with greater values indicating more venues and more people covered by 100% smoke-free laws in a county. (For example, the score of 1 means that all four venues and all people living in the county are covered by 100% smoke-free laws.) Similarly, the smoke-free bar law coverage score (also a continuous variable ranging from 0 to 1) captured the proportion of population covered by 100% smoke-free bar laws within each county.

Current smokers were defined as smoking ≥100 cigarettes in their lifetime and smoking “every day” or “some days” now. Non-smokers included both never smokers (who smoked <100 cigarettes in their lifetime) and former smokers (who had smoked ≥100 cigarettes in lifetime but smoked “not at all” now). Sociodemographic variables included gender, age (18–20, 21–24, 25–44, 45–64, and 65 and older), race/ethnicity (non-Hispanic White, non-Hispanic Black, non-Hispanic other, and Hispanic), education attainment (less than high school, high school graduate or general equivalency diploma (GED), some college, and college graduate), and poverty status (poor, near poor, not poor, and unreported). Following the US Census Bureau*’*s definition [47], poverty status was expressed as a ratio of family income to the appropriate poverty threshold (given family size and number of children). “Poor” people had a family income below the poverty threshold, “near poor” had a family income of 100%–199% of the poverty threshold, and “not poor” had a family income of ≥200% of the poverty threshold.

### 2.4. Statistical Analysis

We had no direct access to the restricted NHIS data, because the FIPS county code is a restricted identification variable. Data were accessed through the CDC Research Data Center (RDC) [48]. We used the remote access system (ANDRE) to analyze data by submitting SAS^®^ software programming code through ANDRE and receiving output via e-mail. Data were weighted to account for the complex sample design and to adjust for non-response and post-stratification [36]. We used descriptive statistics to calculate the weighted proportion of hazardous drinking outcomes (i.e., heavy drinking and binge drinking) by gender, age group, race/ethnicity, education, poverty status and current smoking status. Chi-square tests were conducted to compare the difference in each hazardous drinking outcome by covariates.

Separate multivariable logistic regression models were used to examine if smoke-free law coverage and smoke-free bar law coverage were associated with hazardous drinking outcomes among drinkers (*n* = 17,057), controlling for sociodemographic variables and current smoking status. We used multivariable logistic regression models among a subgroup of drinkers who reported current smoking (*n* = 4074) to examine the association between smoke-free law (and bar law) coverage and hazardous drinking controlling for sociodemographic variables.

We calculated the variance inflation factors (VIF) for all models, and the VIFs were between 1.012 and 1.192 indicating that multicollinearity was not an issue. The interaction of smoke-free law (or bar law) coverage score and current smoking were tested in the analysis among drinkers, and the interactions were not significant (*p* > 0.297 in all cases). Thus, findings reported in this study were the regression models without the interactions. We also tested the multivariable logistic regression models with state and region variables (coded as “northeast”, “midwest”, “south”, or “west”), and found that state and region did not change the association between smoke-free law coverage and hazardous drinking (significance level and direction). Therefore, we excluded the state and region variables in the final models. More details about calculating smoke-free law coverage score, merging data sets, data processing and model formulae can be found in Supplementary Table S1.

## 3. Results

Table 1 shows the characteristics of U.S. adult drinkers. Overall, 8.2% of drinkers reported heavy drinking, and 35.6% reported binge drinking. Around 3.4% drinkers were under the minimum legal drinking age of 21. Heavy drinking was more common among young adults aged 21–24 years (*p* < 0.001), non-Hispanic whites (*p* < 0.001), people who did not graduate from high school (*p* < 0.001), those who were poor and near poor (*p* = 0.003), and current smokers (*p* < 0.001). Males and females were not statistically different in heavy drinking (8.7% vs. 7.7%; *p* = 0.057). Binge drinking was more common among men (*p* < 0.001), adults aged 21–24 years (*p* < 0.001), Hispanics and non-Hispanic whites (*p* < 0.001), people with less than high school education (*p* < 0.001), people who were poor and near poor (*p* < 0.001), and current smokers (*p* < 0.001). Over half (53.0%) of underage drinkers (aged 18–20) reported binge drinking.

Table 2 shows the multivariable logistic regression models that analyzed the association between smoke-free law coverage and hazardous drinking outcomes among drinkers. Smoke-free law coverage was not associated with heavy drinking (adjusted odds ratio (AOR) = 1.22, 95% confidence interval (CI) = 0.99–1.50, *p* = 0.055) or binge drinking (AOR = 1.09, 95% CI = 0.93–1.26, *p* = 0.281) controlling for sociodemographic factors and current smoking status. Young adults aged 18–20 years were less likely to report heavy drinking than adults aged 45–64 years (AOR = 0.49, 95% CI = 0.32–0.75, *p* = 0.002), but were more likely to report binge drinking (AOR = 3.05, 95% CI = 2.29–4.06, *p* < 0.001). When substituting smoke-free bar law coverage for smoke-free law coverage, we found that smoke-free bar law coverage was not associated with heavy drinking (AOR = 1.14, 95% CI = 0.96–1.35, *p* = 0.127) or binge drinking (AOR = 1.11, 95% CI = 0.99–1.25, *p* = 0.083) among all drinkers (Supplementary Table S2).

Subset analyses among drinkers who reported current smoking showed that smoke-free law coverage was unrelated to heavy drinking (AOR = 1.11, 95% CI = 0.84–1.48, *p* = 0.453) or binge drinking (AOR = 1.14, 95% CI = 0.89–1.46, *p* = 0.294) controlling for sociodemographic variables (Supplementary Table S3). Also, smoke-free bar law coverage was not associated with heavy drinking (AOR = 1.03, 95% CI = 0.82–1.29, *p* = 0.782) or binge drinking (AOR = 1.12, 95% CI = 0.93–1.35, *p* = 0.248; Supplementary Table S4).

## 4. Discussion

This study is among the few that examined the association between smoke-free law coverage and hazardous drinking behaviors in a large population-based sample of U.S. adults. Unlike the previous studies which only examined smoke-free bar and/or restaurant laws or statewide smoke-free laws, we assessed local-level smoke-free laws and accounted for the various combinations of law coverage in four major venues (e.g., public workplaces, private workplaces, restaurants and bars) as well as the proportion of the county population covered by these laws. Thus, our measure of smoke-free law coverage captured both the dimension and the breadth of the laws.

Smoke-free law (and bar law) coverage was not associated with hazardous drinking. Among the limited research about the effect of smoke-free laws on alcohol consumption , Young-Wolff et al. [31] found that statewide smoke-free bar and restaurant laws increased AUD remission overall and reduced AUD onset among females in a longitudinal study. McKee et al. [29] compared alcohol consumption quantity between adults in Scotland who experienced the implementation of smoke-free bar laws and those in the other places of the UK who experienced no policy change. Results indicated that there was no difference in drinking quantity change between the two groups pre- and post-legislation; however, there was a significant reduction in drinking quantity among moderate drinkers in Scotland. Krauss et al. [32] found that, for each 1 point increase in statewide smoke-free law coverage index, the state per capita total alcohol consumption dropped 1.1% (primarily for spirits consumption). Picone et al. [33] conducted a study among people aged 51 years and above, and found that greater statewide smoke-free law coverage was associated with reduced drinking quantity among females only. The limited evidence regarding the relationship between smoke-free laws and alcohol use suggested that smoke-free laws might have different effects on alcohol consumption for different subgroups. Longitudinal research is warranted to investigate how smoke-free laws would affect alcohol use patterns and alcohol-related mortality and morbidity by gender, race/ethnicity, age group, smoking status, alcohol consumption level, and/or other psychosocial characteristics.

Subgroup analysis revealed that there was no relationship between smoke-free law (and bar law) coverage and hazardous drinking among drinkers who smoked. Although we used a cross-sectional design, our finding was consistent with a longitudinal study that compared alcohol consumption between smokers who experienced smoke-free bar law implementation and other smokers who experienced no change in the law. Kasza et al. [30] found that the smoke-free bar law had no effect on drinking outcomes overall, but that the law was associated with lower drinking frequency among heavy smokers and a small reduction in drinking among smokers who reported heavy or binge drinking. It could be that smoke-free laws primarily affect at-risk subgroups such as hazardous drinkers, but the reduction in drinking quantity among these hazardous drinkers is not large enough to convert them from hazardous drinkers to non-hazardous drinkers. Therefore, we found no relationship between smoke-free law coverage and hazardous drinking outcomes. Future research should take into account drinking and smoking patterns while assessing the effect of smoke-free laws on alcohol consumption.

This study had several limitations. First, we used one wave of cross-sectional data. The study design did not allow causal conclusions on the relationship between smoke-free law coverage and hazardous drinking outcomes. Second, the study was based on self-reports, and thus was subject to recall and reporting bias. Third, our regression models were conducted based on the assumption that the relationship between independent variables and the logit of hazardous drinking was linear. A nonlinear relationship might exist between independent variables and outcomes. Fourth, our models did not control for all the factors that might affect alcohol use, such as alcohol tax and other alcohol-related policies. Thus, the result might under- or over-estimate the relationship between smoke-free law coverage and hazardous drinking outcomes. We did, however, assess logistic regression models that included state and region variables (both of which reflect alcohol policies and price, and drinking culture in the state and the region) and found that state and region did not change the association between smoke-free law coverage and hazardous drinking (significance level and direction). Fifth, there is no consensus on the definition of binge drinking. The NHIS used a gender-universal cutoff value to define binge drinking (≥5 drinks on at least one day during the past year). While the NIAAA used gender-specific cutoff values to define binge drinking (≥5 drinks for men and ≥4 drinks for women in about 2 h). Thus, the result might under- or over-estimated the binge drinking outcome.

More longitudinal studies are needed to examine if and how county-level smoke-free laws affect alcohol use and other tobacco-related risky behaviors. It was also noteworthy that we defined heavy drinking as 14 drinks per week for men and 7 drinks per week for women, and defined binge drinking as having 5 or more drinks on at least one day in the past 12 months. For countries using other definitions for hazardous drinking outcomes, they may observe a different relationship between smoke-free laws and alcohol consumption. Future research that examines the impact of smoke-free laws on alcohol use should clarify how the outcomes of interest are defined.

## 5. Conclusions

This is among the few studies that have examined the association between smoke-free laws and alcohol use among U.S. adults. Despite the limitations of using a cross-sectional design, our study contributes to the body of literature that asserts that smoke-free law and bar law coverages are not associated with hazardous drinking outcomes. It provides further evidence that policymakers can implement smoke-free laws to protect nonsmokers from secondhand smoke exposure (which contribute to reducing smoking) without increasing risk of alcohol-related health issues among drinkers.

## Figures and Tables

**Table 1:** Sample characteristics of U.S. adult drinkers in 2009, and the comparisons of hazardous drinking by sociodemographic factors and current smoking status.

	Total		Heavy Drinking ^b^			Binge Drinking ^c^		
	*n*	(% ^a^)	*N*	(% ^a^)	*p*	*N*	(% ^a^)	*p*
Overall	17,057	(100)	1402	(8.2)		5746	(35.6)	
Gender					0.057			<0.001
Male	8481	(53.0)	755	(8.7)		3773	(46.2)	
Female	8576	(47.0)	647	(7.7)		1973	(23.8)	
Age group (years)					<0.001			<0.001
18–20	459	(3.4)	34	(5.5)		242	(53.0)	
21–24	1276	(8.7)	153	(11.8)		722	(59.6)	
25–44	7026	(40.5)	528	(7.4)		2889	(42.6)	
45–64	5972	(35.4)	514	(8.8)		1641	(28.4)	
65 and above	2324	(12.0)	173	(7.5)		252	(11.1)	
Race/ethnicity					<0.001			<0.001
White non-Hispanic	11,055	(73.9)	1024	(9.0)		3834	(36.6)	
Black non-Hispanic	2346	(10.0)	153	(6.3)		598	(27.6)	
Other non-Hispanic	873	(4.1)	49	(5.4)		250	(28.7)	
Hispanic	2783	(12.1)	176	(5.8)		1064	(38.6)	
Education					<0.001			<0.001
Less than high school	1965	(10.2)	200	(11.3)		716	(39.4)	
High school graduate/GED	4239	(25.3)	400	(9.3)		1474	(36.3)	
Some college/associate degree	5479	(32.4)	442	(8.2)		1913	(37.3)	
Bachelor’s degree or advanced	5323	(32.1)	354	(6.4)		1628	(32.1)	
Poverty status ^d^					0.003			<0.001
Poor	2103	(9.3)	204	(10.6)		837	(41.2)	
Near poor	2424	(12.6)	231	(10.1)		901	(40.2)	
Not poor	10,880	(67.7)	849	(7.7)		3586	(34.8)	
Unspecified	1650	(10.5)	118	(7.6)		422	(30.1)	
Current smoking ^e^					<0.001			<0.001
No	12,963	(76.2)	758	(5.7)		3773	(30.5)	
Yes	4074	(23.8)	642	(16.2)		1967	(52.4)	

^a^ Weighted percentage; ^b^ Heavy drinking was defined as >14 drinks per week for men and >7 drinks per week for women; ^c^ Binge drinking was defined as ≥5 drinks on at least one day in the past year; ^d^ Poverty status is a ratio of family income to the appropriate poverty threshold (given family size and number of children) defined by the U.S. Census Bureau. “Poor” people had a family income below the poverty threshold, “near poor” had a family income of 100%–199% of the poverty threshold, and “not poor” reported a family income of ≥200% of the poverty threshold; ^e^ Current smoking was defined as smoking ≥100 cigarettes in lifetime and smoking “every day” or “some days” now.

**Table 2:** Association between smoke-free law coverage and hazardous drinking among drinkers.

	Heavy Drinking ^a^		Binge Drinking ^b^	
	Adjusted OR ^c^ [95% CI]		Adjusted OR ^c^ [95% CI]	
Smoke-free law coverage score (ranging between 0 and 1)	1.22	[0.99, 1.50]	1.09	[0.93, 1.26]
Female	0.92	[0.80, 1.05]	0.33 ***	[0.30, 0.36]
Age group (years)				
18–20	0.49 **	[0.32, 0.75]	3.05 ***	[2.29, 4.06]
21–24	1.28	[0.96, 1.70]	4.25 ***	[3.43, 5.25]
25–44	0.81 *	[0.69, 0.95]	1.97 ***	[1.77, 2.20]
45–64	1.00		1.00	
65 and above	0.93	[0.74, 1.15]	0.32 ***	[0.26, 0.38]
Race/ethnicity				
White non-Hispanic	1.00		1.00	
Black non-Hispanic	0.61 ***	[0.47, 0.78]	0.51 ***	[0.44, 0.61]
Others non-Hispanic	0.59 **	[0.40, 0.87]	0.54 ***	[0.43, 0.68]
Hispanic	0.59 ***	[0.46, 0.75]	0.81 **	[0.71, 0.93]
Education				
Less than high school	1.40 *	[1.08, 1.82]	0.97	[0.80, 1.17]
High school graduate/GED	1.09	[0.89, 1.33]	0.97	[0.85, 1.10]
Some college/associate degree	1.08	[0.90, 1.31]	1.08	[0.96, 1.21]
Bachelor’s degree or advanced	1.00		1.00	
Poverty status ^d^				
Poor	1.21	[0.96, 1.52]	0.96	[0.82, 1.13]
Near poor	1.19	[0.91, 1.54]	1.08	[0.92, 1.27]
Not poor	1.00		1.00	
Unspecified	0.94	[0.72, 1.24]	0.87	[0.72, 1.05]
Current smoking ^e^				
No	1.00		1.00	
Yes	3.00 ***	[2.60, 3.47]	2.27 ***	[2.02, 2.54]
Fit statistics				
*N*	16,895		16,734	
Design df	300		300	
F(16, 285)	15.94		121.23	
P	<0.00005		<0.0005	
R^2^	0.033		0.161	

OR = odds ratio; CI = confidence interval; ^a^ Heavy drinking was defined as >14 drinks per week for men and >7 drinks per week for women; ^b^ Binge drinking was defined as ≥5 drinks on at least one day in the past year; ^c^ Multivariable logistic regression models controlled for all variables listed in the table; ^d^ Poverty status is a ratio of family income to the appropriate poverty threshold (given family size and number of children) defined by the US Census Bureau. “Poor” people had a family income below the poverty threshold, “near poor” had a family income of 100%–199% of the poverty threshold, and “not poor” reported a family income of ≥200% of the poverty threshold; ^e^ Current smoking was defined as smoking ≥100 cigarettes in lifetime and smoking “every day” or “some days” now; * *p* < 0.05; ** *p* < 0.01; *** *p* < 0.001.

## Supplementary Materials

Supplementary materials can be found at www.mdpi.com/1660-4601/14/4/412/s1. Table S1. Supplementary information on methods. Table S2. Association between smoke-free bar law coverage and hazardous drinking among drinkers. Table S3. Association between smoke-free law coverage and hazardous drinking among drinkers who reported current smoking. Table S4. Association between smoke-free bar law coverage and hazardous drinking among drinkers who reported current smoking.
