# Supplementary material for: Smoke-Free Laws and Hazardous Drinking: A Cross-Sectional Study among U.S. Adults
Source: Int J Environ Res Public Health. 2017 Apr 13;14(4):412. doi: 10.3390/ijerph14040412 (PMC5409613; doi:10.3390/ijerph14040412)
Supplement: Supplementary file 1 [file ijerph-14-00412-s001.pdf]

Smoke-free Laws and Hazardous Drinking: A Cross-Sectional Study among U.S. Adults

Nan Jiang, Mariaelena Gonzalez, Pamela M. Ling, Kelly C. Young-Wolff and Stanton A. Glantz

Supplementary Materials

Table S1. Supplementary information on methods.

1. Calculation of 100% smoke-free law coverage score

We combined 2009 U.S. Census Population Estimates dataset and 2009 American Nonsmokers' Rights Foundation (ANRF) U.S. Tobacco Control Laws Database to calculate 100% smoke-free law coverage score.

1.1. U.S. Census Population Estimates

The 2009 U.S. Census Population Estimates dataset provides population estimates for all incorporated places (self-governing cities, towns, villages, or boroughs) and for all census designated places (statistical counterparts of incorporated places, geographic areas having settled concentrations of population without municipal organization or official political designation) from 2000 to 2009 within the United States, except for Indian reservations and tribal-owned land.

1.2. ANRF U.S. Tobacco Control Laws Database

The ANRF U.S. Tobacco Control Laws Database is a national collection of local tobacco control ordinances and board of health regulations. A 100% smoke-free law is defined by ANRF as a law that prohibits indoor smoking with no exceptions (e.g., smoking rooms or venue-size exceptions). For the purpose of this study, we only kept relevant variables such as (1) the 100% smoke-free laws in each of the four venues (i.e., private and public workplaces, restaurants and bars), (2) the type of 100% smoke-free laws (i.e., 100% smoke-free state law, 100% smoke-free county law and county jurisdiction, and 100% smoke-free sub-county law), and (3) the month and year that the 100% smoke-free laws came in effect.

1.3. Merging Census Population Estimates data and ANRF Tobacco Control Law Database

We merged the Census Population Estimates data and ANRF Tobacco Control Law Database by year and Federal Information Processing Standards (FIPS) county code, a code that consists of two digits of state code and three digits of county code within the state. An FIPS code provides a unique identification of counties and county equivalents in the U.S.

1.4. 100% smoke-free law coverage score

First, we calculated the number of people covered by a 100% smoke-free law in each of the four venues (i.e., private and public workplaces, restaurants and bars) within each county using the algorithm as below:

• 100% smoke-free state law	Number of people covered by smoke-free law in a county = Total county population
• 100% smoke-free county law with a county jurisdiction of the entire county area	Number of people covered by smoke-free law in a county = Total county population
• 100% smoke-free county law with a county jurisdiction of unincorporated areas only	Number of people covered by smoke-free law in a county = (Unincorporated population) + (Incorporated population covered by a 100% smoke-free sub-county law)
• No 100% smoke-free state or county law	Number of people covered by smoke-free law in a county = (Unincorporated population covered by a 100% smoke-free sub-county law) + (Incorporated population covered by a 100% smoke-free sub-county law)

Second, for each county, we divided the number of people covered by a 100% smoke-free law in each of the four venues within the county by the total county population to obtain four ratios, which indicated the proportion of county population covered by a 100% smoke-free law in each venue. The ratio was a continuous variable ranging from 0 to 1. The greater the ratio, the more county population covered by a 100% smoke-free law.

Third, we took the mean of the above four ratios within each county to obtain a score, which represented the proportion of county population covered by 100% smoke-free laws in all four venues. This score was defined 100% smoke-free law score in our study. The smoke-free law coverage score was a continuous variable ranging from 0 to 1, and it accounted for the number of venues covered by a 100% smoke-free law and the proportion of county population covered by 100% smoke-free laws. A greater score meant that more venues and more people were covered by 100% smoke-free laws in a county. (For example, the score of 1 meant that all four venues and all of the county population were covered by 100% smoke-free laws.)

2. Remote data access through Research Data Center

To link the smoke-free law coverage scores to alcohol consumption measures, we merged ANRF U.S. Tobacco Control Ordinance Database with 2009 National Health Interview Survey (NHIS) restricted data set by FIPS county code. FIPS code is a restricted identification variable. Therefore, we had no direct access to the data which are hosted by the Centers for Disease Control and Prevention's Research Data Center (RDC). We sent the ANRF data (with FIPS county code and 100% smoke-free law coverage scores) to a RDC analyst who merged the ANRF dataset with NHIS restricted database. Then we accessed data through RDC's remote access system (ANDRE) by remotely submitting SAS® software programming code to the automated ANDRE and receiving output via e-mail.

3. Statistical analyses

We conducted data analyses from December 2013 to January 2014. We remotely submitted SAS programming code to ANDRE and received output via e-mail. Data were weighted following the instruction of 2006-2012 NHIS Survey Description Documents to account for complex sample design and adjust for non-response and post-stratification. We set up the weights by the syntax "svyset [pw= wtfa_sa], strata (strat_p) psu (psu_p)" and used "svy" comments in all analyses.

3.1. Descriptive analyses

We used descriptive statistics to calculate weighted prevalence of the two hazardous drinking outcomes (i.e., heavy drinking and binge drinking) by gender, age group, race/ethnicity, education, poverty status and current smoking status among current alcohol drinkers (n = 17,057). Chi-square tests were conducted to compare the difference in each hazardous drinking outcome by gender, age group, race/ethnicity, education, poverty status and current smoking status. Findings from Chi-square tests showed that all the covariates were related to the two hazardous drinking outcomes, except for the relationship between heavy drinking and gender ($p = 0.057$).

3.2. Association between 100% smoke-free law coverage score and hazardous drinking

Based on the Chi-square findings, we conducted separate multivariate logistic regression models among all alcohol users to examine the relationship of hazardous drinking outcomes with 100% smoke-free law coverage score, controlling for gender, age group, race/ethnicity, education, poverty status and current smoking status. Previous studies (as below) examining the association between smoke-free laws and alcohol use also controlled these variables in the adjusted models.

McKee SA, Higbee C, O'Malley S, Hassan L, Borland R, et al. Longitudinal evaluation of smoke-free Scotland on pub and home drinking behavior: Findings from the International Tobacco Control Policy Evaluation Project. Nicotine & Tobacco Research. 2009;11: 619-626.

Kasza KA, McKee SA, Rivard C, Hyland AJ. Smoke-free bar policies and smokers' alcohol consumption: findings from the International Tobacco Control four country survey. Drug and Alcohol Dependence. 2012;126: 240-245.

Young-Wolff KC, Hyland AJ, Desai R, Sindelar J, Pilver CE, et al. Smoke-free policies in drinking venues predict transitions in alcohol use disorders in a longitudinal U.S. sample. Drug and Alcohol Dependence. 2013;128: 214-221.

We did not control for alcohol policies (e.g., taxation and pricing) in the final regression model, because those data were not available to us when we conducted data analyses. However, we did test the model with geographic variables, including state and region (coded as "northeast", "midwest", "south", or "west"). State and region variables reflected the drinking culture and alcohol taxation and pricing in the area. We found that state and region did not change the association between smoke-free law coverage score and hazardous drinking (significance level and direction). Therefore, we excluded the geographic variables in the multivariate regression models.

3.3. Association between 100% smoke-free bar law coverage and hazardous drinking

We repeated the analyses in the Section 3.2. and used 100% smoke-free bar law coverage score to replace the 100% smoke-free law coverage score in order to assess the relationship between smoke-free bar law coverage and hazardous drinking among alcohol drinkers.

3.4. Subset analyses of the association between 100% smoke-free law coverage and hazardous drinking

Tobacco and alcohol co-use is common, and co-users represent a high-risk group. It is important to explore if smoke-free law coverage is related to hazardous drinking among tobacco-and-alcohol co-users. Therefore, we conducted subset analyses among alcohol drinkers who reported current cigarette smoking (n=4,074). We conducted separate multivariate logistic regression models to examine the association between 100% smoke-free law (and bar law) coverage scores and the two hazardous drinking outcomes, controlling for gender, age group, race/ethnicity, education and poverty status.

3.5. Multicollinearity

We calculated the variance inflation factors (VIF) for all our models. The VIFs were between 1.012 and 1.192, indicating that multicollinearity was not an issue.

3.6. Interactions

We tested the interactions of 100% smoke-free law coverage score and smoking status, and found that the interactions were not significant in all cases. Therefore, we excluded the interactions and reported the main effect of the smoke-free law coverage in the final models.

Table S2. Association between smoke-free bar law coverage and hazardous drinking among drinkers.

	Heavy drinking [a]	Binge drinking [b]
	Adjusted OR [c] [95% CI]	Adjusted OR [c] [95% CI]
Smoke-free bar law coverage score (ranging between 0 and 1)	1.14 [0.96, 1.35]	1.11 [0.99, 1.25]
Female	0.92 [0.80, 1.06]	0.33 [0.30, 0.36] ***
Age group (years)		
18–20	0.49 [0.32, 0.75] **	3.04 [2.28, 4.05] ***
21–24	1.28 [0.96, 1.70]	4.25 [3.43, 5.25] ***
25–44	0.81 [0.69, 0.95] *	1.97 [1.77, 2.20] ***
45–64	1.00	1.00
65 and above	0.93 [0.75, 1.16]	0.32 [0.26, 0.38] ***
Race/ethnicity		
White non-Hispanic	1.00	1.00
Black non-Hispanic	0.61 [0.48, 0.78] ***	0.52 [0.44, 0.61] ***
Others non-Hispanic	0.59 [0.40, 0.87] **	0.54 [0.43, 0.67] ***
Hispanic	0.58 [0.46, 0.75] ***	0.80 [0.70, 0.92] **
Education		
Less than high school	1.40 [1.08, 1.81] *	0.97 [0.80, 1.18]
High school graduate/GED	1.09 [0.89, 1.33]	0.97 [0.86, 1.10]
Some college/associate degree	1.08 [0.89, 1.31]	1.08 [0.96, 1.21]
Bachelor's degree or advanced	1.00	1.00
Poverty status [d]		
Poor	1.21 [0.96, 1.52]	0.97 [0.83, 1.13]
Near poor	1.19 [0.91, 1.55]	1.08 [0.92, 1.27]
Not poor	1.00	1.00
Unspecified	0.95 [0.72, 1.24]	0.87 [0.72, 1.05]
Current smoking [e]		
No	1.00	1.00
Yes	3.00 [2.60, 3.47] ***	2.27 [2.02, 2.55] ***
Fit statistics		
n	16,895	16,734
Design df	300	300
$F(16, 285)$	16.07	121.16
P	<.00005	<.0005
R^2	0.033	0.161

Notes. OR = odds ratio; CI = confidence interval.

[a] Heavy drinking was defined as >14 drinks per week for men and >7 drinks per week for women.

[b] Binge drinking was defined as ≥5 drinks on at least one day in the past year.

[c] Multivariable logistic regression models controlled for all variables listed in the table.

[d] Poverty status is a ratio of family income to the appropriate poverty threshold (given family size and number of children) defined by the US Census Bureau. "Poor" people had a family income below the poverty threshold, "near poor" had a family income of 100–199% of the poverty threshold, and "not poor" reported a family income of ≥200% of the poverty threshold.

[e] Current smoking was defined as smoking ≥100 cigarettes in lifetime and smoking "every day" or "some days" now.

* $p < 0.05$; ** $p < 0.01$; *** $p < 0.001$.

Table S3. Association between smoke-free law coverage and hazardous drinking among drinkers who reported current smoking.

	Heavy drinking [a]	Binge drinking [b]
	Adjusted OR [c] [95% CI]	Adjusted OR [c] [95% CI]
Smoke-free law coverage score (ranging between 0 and 1)	1.11 [0.84, 1.48]	1.14 [0.89, 1.46]
Female	0.74 [0.59, 0.93] *	0.35 [0.29, 0.42] ***
Age group (years)		
18–20	0.54 [0.30, 0.98] *	2.00 [1.21, 3.32] **
21–24	1.25 [0.81, 1.93]	3.14 [1.99, 4.95] ***
25–44	0.79 [0.62, 1.00]	1.86 [1.53, 2.26] ***
45–64	1.00	1.00
65 and above	0.80 [0.53, 1.19]	0.22 [0.14, 0.35] ***
Race/ethnicity		
White non-Hispanic	1.00	1.00
Black non-Hispanic	0.78 [0.53, 1.14]	0.70 [0.53, 0.93] *
Others non-Hispanic	0.72 [0.38, 1.36]	0.62 [0.39, 0.97] *
Hispanic	0.61 [0.42, 0.88] **	0.90 [0.69, 1.17]
Education		
Less than high school	1.12 [0.74, 1.70]	0.64 [0.46, 0.88] **
High school graduate/GED	0.96 [0.69, 1.33]	0.81 [0.62, 1.05]
Some college/associate degree	0.82 [0.57, 1.19]	0.79 [0.61, 1.02]
Bachelor's degree or advanced	1.00	1.00
Poverty status [d]		
Poor	1.07 [0.78, 1.47]	0.81 [0.64, 1.03]
Near poor	0.98 [0.72, 1.35]	0.95 [0.75, 1.19]
Not poor	1.00	1.00
Unspecified	1.16 [0.75, 1.78]	0.83 [0.55, 1.26]
Fit statistics		
n	4,019	3,938
Design df	300	300
F(16, 285)	1.93	32.22
P	.020	<.0005
R^2	0.012	0.125

Notes. OR = odds ratio; CI = confidence interval.

[a] Heavy drinking was defined as >14 drinks per week for men and >7 drinks per week for women.

[b] Binge drinking was defined as ≥5 drinks on at least one day in the past year.

[c] Multivariable logistic regression models controlled for all variables listed in the table.

[d] Poverty status is a ratio of family income to the appropriate poverty threshold (given family size and number of children) defined by the US Census Bureau. "Poor" people had a family income below the poverty threshold, "near poor" had a family income of 100–199% of the poverty threshold, and "not poor" reported a family income of ≥200% of the poverty threshold.

* $p < 0.05$; ** $p < 0.01$; *** $p < 0.001$.

Table S4. Association between smoke-free bar law coverage and hazardous drinking among drinkers who reported current smoking.

	Heavy drinking [a]	Binge drinking [b]
	Adjusted OR [c] [95% CI]	Adjusted OR [c] [95% CI]
Smoke-free bar law coverage score (ranging between 0 and 1)	1.03 [0.82, 1.29]	1.12 [0.93, 1.35]
Female	0.74 [0.59, 0.93] *	0.35 [0.29, 0.42] ***
Age group (years)		
18–20	0.54 [0.30, 0.98] *	2.00 [1.20, 3.31] **
21–24	1.25 [0.81, 1.93]	3.14 [1.99, 4.96] ***
25–44	0.79 [0.62, 1.00]	1.86 [1.53, 2.26] ***
45–64	1.00	1.00
65 and above	0.80 [0.53, 1.19]	0.22 [0.15, 0.35] ***
Race/ethnicity		
White non-Hispanic	1.00	1.00
Black non-Hispanic	0.77 [0.53, 1.14]	0.70 [0.53, 0.93] *
Others non-Hispanic	0.72 [0.38, 1.36]	0.61 [0.39, 0.97] *
Hispanic	0.61 [0.42, 0.88] **	0.89 [0.69, 1.16]
Education		
Less than high school	1.12 [0.74, 1.70]	0.64 [0.46, 0.88] **
High school graduate/GED	0.96 [0.69, 1.33]	0.81 [0.62, 1.05]
Some college/associate degree	0.82 [0.57, 1.19]	0.79 [0.61, 1.02]
Bachelor's degree or advanced	1.00	1.00
Poverty status [d]		
Poor	1.07 [0.78, 1.46]	0.81 [0.64, 1.03]
Near poor	0.98 [0.71, 1.34]	0.95 [0.75, 1.19]
Not poor	1.00	1.00
Unspecified	1.16 [0.76, 1.79]	0.83 [0.55, 1.26]
Fit statistics		
n	4,019	3,938
Design df	300	300
F(16, 285)	1.99	32.31
P	.016	<.0005
R^2	0.012	0.125

Notes. OR = odds ratio; CI = confidence interval.

[a] Heavy drinking was defined as >14 drinks per week for men and >7 drinks per week for women.

[b] Binge drinking was defined as ≥5 drinks on at least one day in the past year.

[c] Multivariable logistic regression models controlled for all variables listed in the table.

[d] Poverty status is a ratio of family income to the appropriate poverty threshold (given family size and number of children) defined by the US Census Bureau. "Poor" people had a family income below the poverty threshold, "near poor" had a family income of 100–199% of the poverty threshold, and "not poor" reported a family income of ≥200% of the poverty threshold.

$^{*}p < 0.05$; $^{**}p < 0.01$; $^{***}p < 0.001$.
